# Efficacy of Jintiange Capsules in the Treatment of Osteoporosis: A Network Meta‐analysis

**DOI:** 10.1111/os.12439

**Published:** 2019-03-10

**Authors:** Jing Sun, Xiong‐gang Yang, Yong‐cheng Hu

**Affiliations:** ^1^ Editorial Office of Orthopaedic Surgery Tianjin Hospital Tianjin China; ^2^ Graduate School of Tianjin Medical University Tianjin China; ^3^ Department of Bone Tumor Tianjin Hospital Tianjin China

**Keywords:** Efficacy, Jintiange capsule, NMA (network meta‐analysis), Osteoporosis

## Abstract

**Objective:**

To evaluate the efficacy of Jintiange capsules and Jintiange combined with other therapies in the treatment of osteoporosis.

**Methods:**

A systematic review of the literature was conducted through databases including China National Knowledge Infrastructure (CNKI), the VIP Database for Chinese Technical Periodicals (VIP), Wanfang, and PubMed from inception to April 2018. Network meta‐analysis was used to determine the relative efficacy of related treatments on osteoporosis. The primary outcome measures are the bone mineral density (BMD) of the lumbar and femoral neck, and the secondary outcome measures are visual analog pain score (VAS) and adverse events. Two reviewers independently selected the studies, extracted information, and assessed the quality of included trials. Data extracted from eligible studies was pooled and meta‐analyzed, and the mean differences (MD) with their 95% confidence intervals were estimated as the effect size between treatments.

**Results:**

Thirty‐one studies were included in this study, containing 28 randomized controlled trials (RCT) and 3 non‐randomized controlled trials (non‐RCT), with a total of 14 regimens treating osteoporosis. According to the surface under the cumulative ranking (SUCRA) curves, Jintiange capsules combined with atorvastatin (89.9%) and Jintiange combined with bisphosphonates (88.2%) have the best efficacy in terms of the BMD of the lumbar and femoral neck, respectively. Based on the VAS, Jintiange combined with calcium has the best analgesic effect (83.4%).

**Conclusion:**

Jintiange capsules alone and combined with other therapies is a good choice for treating patients with osteoporosis in terms of improving BMD, relieving pain, and reducing adverse events. More large‐scale and well‐designed RCT are warranted to confirm the results of this study.

## Introduction

Osteoporosis, a systemic metabolic disease that reduces bone mass and impairs bone microarchitecture, can cause bone pain and increase individuals’ susceptibility to fragility fractures^1^. The prescribed treatments for osteoporosis mainly include two classes: basic drugs and anti‐osteoporosis drugs^2^. The former consists of calcium and vitamin D, and the latter refers to bone absorption‐inhibitor drugs (i.e. bisphosphonates [BP], calcitonin, and estrogen) and bone formation‐acceleration drugs (i.e. RABKL inhibitor and traditional Chinese medicine [TCM]). TCM is widely used in the treatment of osteoporosis. As a new first‐class drug, the efficacy of Jintiange capsules in the treatment of osteoporosis is particularly outstanding. Jintiange capsules are composed of artificial tiger bone powder, which is made using different animal bones. The constituents and effect are very similar to tiger bone, the use of which is prohibited because tigers are a protected animal in China.

In both TCM and Western medicine theory, tiger bone is understood to have significant effects on strengthening bone and relieving pain. Jintiange capsules have been proven to contain a high level of calcium and the ratio of calcium and phosphorus within the capsules means that they are suitable for the human body to absorb^3^, ^4^, ^5^. They also contain multiple organic ingredients, such as collagen, bone morphogenetic protein, bone growth factors, and polysaccharide, which can accelerate bone formation, inhibit bone absorption, and improve bone density^6^, ^7^. Studies also show that tiger bone can elevate individuals’ pain threshold and relieve bone pain^3^, ^8^, ^9^, ^10^, ^11^.

Network meta‐analysis (NMA) is a type of meta‐analysis that combines direct and indirect evidence about treatment effects of studies that share at least one treatment in common with at least one other study^12^. NMA provides network diagrams depicting direct and indirect comparisons, and can be used to predict the efficacy rank of different treatments (using a surface under the cumulative ranking graph [SUCRA]). Many studies report the effects of Jintiange capsules or combined therapy in the treatment of osteoporosis; however, analyses are often restricted by the limited amount of data available to compare related treatment methods. Therefore, the aim of this study is to evaluate the comparative effectiveness using an NMA to allow a comprehensive analysis of the evidence related to all relevant treatments for osteoporosis.

## Methods and Material

### *Study Inclusion*

Studies were considered for inclusion if they met the following criteria:

- Participants: People who has been diagnosed with osteoporosis. According to the diagnostic criteria recommended by the WHO, individuals with bone mineral density (BMD) decreases of less than 1 standard deviation (SD) compared with the bone peak of healthy adults of the same race and gender should be classified as having normal BMD (T score > −1). When the decline is between 1 and 2.5 SD (−2.5 < T score ≤ −1) and more than 2.5 SD, individuals would be diagnosed as having low bone mass and osteoporosis, respectively.
- Interventions and comparisons: At least two study groups with therapy regimens that include Jintiange capsules or Jintiange combined with other treatments.
- Outcome measure: The primary outcome measures are the bone mineral density (BMD) of the lumbar vertebra and femoral neck at final follow up; secondary outcome measures are the visual analog pain score (VAS) as well as the number of adverse events at final follow up.
- Study design: Randomized controlled trails (RCT) and non‐randomized controlled trials (non‐RCT)

Studies were excluded if: (i) they were basic science articles, review articles, news, abstracts, letters, meeting proceedings or academic dissertations; and (ii) contained repeated participants with each other; and (iii) had incomplete data or incorrect data.

### *Search Methods and Study Selection*

To identify studies concerning the efficacy of Jintiange capsules in the treatment of osteoporosis, a systematic literature search was performed. We searched electronic platforms including PubMed, China National Knowledge Infrastructure (CNKI), the VIP Database for Chinese Technical Periodicals (VIP) and the WanFang database from inception to 12 April 2018, and the language was not restricted. Search terms included “Jintiange,” “Jintiange capsule,” “bionic tiger bone”, “artificial tiger bone,” and “osteoporosis” in both English and Chinese. A combined search using the subject terms (Medical Subject Heading, MeSH) and free terms was carried out in PubMed, while only keywords were used for searching in the Chinese databases. Reference lists of the included studies were also viewed for any additional papers. In addition, we consulted related pharmaceutical companies for additional published or unpublished studies.

Two authors independently selected studies following the predetermined selection criteria; any disagreement was resolved by discussion. EndNote X7 17.0 (Clarivate Analytics, Philadelphia, USA) was used to detect and merge the duplicates, and then titles and abstracts were evaluated to identify the ones that met the criteria. Finally, full texts were reviewed for inclusion.

### *Data Extraction*

Two authors independently extracted data from selected studies using data extraction forms, including: (i) publication details (i.e. title, first author, year of publication, and study design); (ii) baseline characteristics (i.e. sample size, gender, and age); (iii) intervention (i.e. selected regimens and administration approaches (including dose, delivery route, interval, and treatment course); and (iv) outcome measures (i.e. BMD of lumbar vertebra and femoral neck, VAS, and number of adverse events). We determined the cause of diversity in obtained information and resolved disagreement through discussion.

### *Assessment for Risk of Bias*

We used the Cochrane risk of bias tool to assess the risk bias of the included studies, and the assessment was performed independently by two authors. The tool includes seven domains: random sequence generation, allocation concealment, blinding of participants and personnel, blinding of outcome assessment, incomplete outcome data, selective reporting, and other bias. The judgment for each domain was a low risk of bias, a high risk of bias, or an unclear risk of bias, and RevMan 5.3 (The Cochrane Collaboration, Oxford, UK) was used for this assessment. For non‐RCT, the methodological index for non‐randomized studies (MINORS) scale was applied for quality assessment, with scores from 0 to 24. Disagreements were resolved by the third reviewer.

### *Statistical Analysis*

The BMD of the lumbar spine and the femoral neck as well as the VAS were all continuous variables. A Bayesian statistics‐based Markov Chain–Monte Carlo (MCMC) random‐effect model was selected to calculate the mean difference (MD) values and 95% confidence intervals (95% CI) between the treatment measures. In addition, if the 95% CI of MD did not include 0 (*P* < 0.05), it was considered statistically significant. We used non‐informative prior distributions and overdispersed initial values in four chains to fit the model, yielding 20 000 iterations (including 5000 tuning iterations) and a thinning interval of 1 for each chain. The calculation of the above model was completed using the computer program of the “GEMTC” installation package in the R 3.5.0 software (R Foundation for Statistical Computing, Vienna, Austria) to invoke the software JAGS 4.2.0 (SourceForge Media, LLC dba Slashdot Media, California, USA).

The network plot, inconsistency detection, the funnel plot and the SUCRA were prepared using Stata 13.0 (StataCorp LLC, College Station, Texas, USA). The inconsistency test evaluates the closed‐loop consistency. When the 95% CI starting point of the inconsistency factor (IF) is “0,” this indicates that the direct and the indirect evidence are consistent. The funnel plot was used to detect the interventions with small sample effects and publication bias. The SUCRA curves were drawn to predict the efficacy of each treatment. The larger the area under the curves (0%–100%), the better the intervention for treatment.

## Results

### *Search Results*

**Figure 1:**
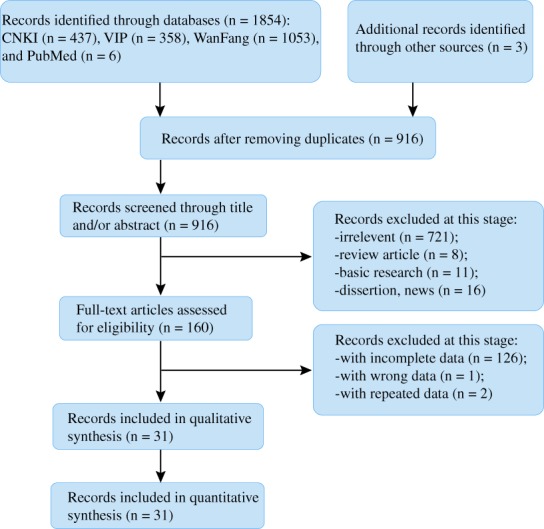
Literature searching process. A total of 1857 articles are identified from databases, including PubMed, China National Knowledge Infrastructure (CNKI), the VIP Database for Chinese Technical Periodicals (VIP), and WanFang database. After removing duplicates and screening all titles and abstracts, 756 articles were excluded. Then full texts were read carefully, and, finally, 31 studies were included.

A total of 1857 potentially relevant articles were identified. After removing duplicates (916 articles) using EndNote X7 and screening all titles and abstracts, 756 articles were excluded. Then full texts were read carefully, and, finally, 31 studies were included: 28 RCT and 3 non‐RCT. All studies are in Chinese. The literature search process is presented in Fig. 1.

### *Study Characteristics*

The 31 studies were published between 2011 to 2018, among which 4 were published in 2017 and 5 were published in 2018^3^, ^10^, ^13^, ^14^, ^15^, ^16^, ^17^, ^18^, ^19^, ^20^, ^21^, ^22^, ^23^, ^24^, ^25^, ^26^, ^27^, ^28^, ^29^, ^30^, ^31^, ^32^, ^33^, ^34^, ^35^, ^36^, ^37^, ^38^, ^39^, ^40^, ^41^. The sample sizes of the 31 studies range from 44 to 867 and include a total of 4330 diagnosed osteoporotic patients, with an age range from 17 to 79 years old. All studies’ reported groups were matched in terms of age, gender, severity, and course of disease. Except for 4 three‐arm experiments, all studies are two‐arm experiments. Twenty‐two studies involve combined therapies, and 9 are single treatment studies (Jintiange capsules only). Among all studies, 22 report on primary osteoporosis (12 postmenopausal osteoporosis and 10 senile osteoporosis cases) and 9 secondary osteoporosis, involving 14 treatments: Jintiange capsules, bisphosphonates, calcium +/VD, atorvastatin, calcitonin, estrogen, physiotherapy, and a combination of these treatments. The dose of Jintiange capsules used is reported to be 1.2 g, 3 times per day in all studies. The average follow‐up time is 6.84 months, with a range from 1 to 16 months. The study characteristics of the included studies are shown in Table 1.

**Table 1:** Characteristics of included studies

No.	Included studies	Treatment group 1			Treatment group 2			Study design	Treatment course (months)
		Treatment	Sample size (male/female)	Age (years)	Treatment	Sample size (Male/Female)	Age (years)		
1	Liu *et al.* (2016)^20^	Jintiange + BP	58 (22/36)	71 ± 5†	BP	58 (24/34)	70 ± 6†	RCT	12
2	Xia & Tang (2013)^10^	Jintiange + calcium	110 (61/49)		Calcium +/VD	120 (62/58)		RCT	12
3	Wang *et al.* (2014)^35^	Jintiange + BP + calcium	43 (19/24)	64 ± 6 †	Calcium +/VD	43 (18/25)	64 ± 6 †	RCT	12
4	Xia & Wang (2015)^31^	Jintiange + calcium	35 (0/35)		Calcium +/VD	35 (0/35)		RCT	16
5	Peng *et al.* (2018)^38^	Jintiange + BP	55 (0/55)		BP	55 (0/55)		Non‐RCT	12
6	Xu *et al.* (2017)^21^	Jintiange + Estrogen	50 (0/50)	61.47 ± 7.38 †	Estrogen	50 (0/50)	61.98 ± 6.92 †	RCT	12
7	Du & Shao (2014)^34^	Jintiange capsule	78	65 (50–73)‡	BP + calcium	78	64 (48–72) ‡	RCT	6
8	Song *et al.* (2016)^17^	Jintiange + atorvastatin	50 (21/29)	65 (51–74) §	Jintiange capsule	50	64 (49–73) §	RCT	6
9	Yu *et al.* (2016)^16^	Jintiange + atorvastatin	30 (12/18)	66 (53–75) ‡	Jintiange capsule	30 (14/16)	64 (51–74) ‡	RCT	6
10	Xie (2018)^24^	Jintiange + BP	446 (0/446)		BP	430 (0/430)		Non‐RCT	3
11	Yeerjiang & Wang (2017)^33^	Jintiange + calcium	100 (53/47)	65.23 ± 11.42 †	Calcium +/VD	100 (52,48)	65.74 ± 11.37 †	RCT	3
12	Fan *et al.* (2015)^28^	Jintiange + calcium	50 (10/40)	62.85 ± 2.25 †	Calcium +/VD	50 (11/39)	62.56 ± 2.73 †	RCT	3
13	Fu *et al.* (2016)^36^	Jintiange + calcium	80 (45/35)	62.7 ± 7.2 †	Calcium +/VD	80 (31/49)	60.2 ± 6.0 †	RCT	3
14	Huang *et al.* (2014)_32_	Jintiange + calcium	84		BP + calcium	82		RCT	3
15	Qin *et al.* (2016)^30^	Jintiange capsule	56 (0/56)	62.5 ± 5.0 †	Calcium +/VD	56 (0/56)	63.6 ± 2.1 †	RCT	3
16	Le (2018)^27^	Jintiange + BP	56 (0/56)	66.79 ± 3.29 †	BP	31 (0/31)	65.68 ± 3.30 †	Non‐RCT	6
17	Xi *et al.* (2016)^37^	Jintiange + BP	84 (40/44)	63.7 ± 7.1 †	BP	83 (41/42)	63.2 ± 6.6 †	RCT	6
18	Liu *et al.* (2018)^22^	Jintiange + BP	76 (0/75)	59.96 ± 9.24 †	BP	75 (0/75)	59.48 ± 9.35 †	RCT	6
19	Luo *et al.* (2015)^15^	Jintiange + BP	23		Calcium +/VD	23		RCT	6
20	Qi *et al.* (2017)^29^	BP + calcium	87 (0/87)	65.3 ± 10.9 †	Jintiange + BP + calcium	46 (0/46)	63.9 ± 14.8 †	RCT	6
21	Zhang *et al.* (2014)^19^	Jintiange + calcitonin	23		Calcitonin	23		RCT	6
22	Dai *et al.* (2018)^26^	Jintiange + BP	80 (0/80)	68.26 ± 5.79 †	BP	80 (0/80)	67.33 ± 6.35 †	RCT	6
23	Gao *et al.* (2016)^3^	Jintiange + BP	53		Jintiange capsule	53		RCT	6
24	Luo *et al.* (2016)^14^	Jintiange + BP + calcium	56		BP + calcium	54		RCT	6
25	Pan *et al.* (2016)^25^	Jintiange + BP	70 (0/70)	67.25 ± 5.43 †	BP	70 (0/70)	67.19 ± 5.40 †	RCT	6
26	Xu *et al.* (2015)^23^	Jintiange + BP + calcium	21	56.3**	calcium +/VD	21	57.1**	RCT	12
27	Fu *et al.* (2017) * ^,39^	Jintiange + BP + calcium	33	67.3 ± 11.2 †	BP + calcium	33	66.9 ± 12.5 †	RCT	12
					calcium +/VD	33	66.6 ± 11.7 †		
28	Du *et al.* (2015) * ^,34^	Jintiange + BP + calcium	34		Jintiange capsule	34		RCT	6
					BP + calcium	34			
29	Wang *et al.* (2014) * ^,18^	Jintiange + physiotherapy + calcium	32		Jintiange + calcium	32		RCT	3
					physiotherapy + calcium	32			
30	Hu *et al.* (2011) * ^,40^	Jintiange + calcium	36		Jintiange capsule	36		RCT	1
					Calcium +/VD	36			
31	Cai *et al.* (2015) * ^,13^	Jintiange + BP	34 (0/34)		Jintiange capsule	32 (0/32)		RCT	6
					BP	32 (0/32)			

Jintiange, Jintiange capsule; BP, bisphosphonate; RCT, randomized controlled trail

* Three‐arm experiment.

† Data are expressed as mean ± SD.

‡ Mean (minimum‐maximum).

§ Median (minimum‐maximum).

** Mean.

### *Risk of Bias in Included Studies*

For the 28 RCT, all studies have definite selection criteria and are described as “randomized,” and 15 studies describe the methods for random sequence generation, such as randomized digital table, stratified randomization, and randomized block. No studies describe adequate allocation concealment or blinding methods. Six studies report the outcome of loss to follow up, among which four had lost cases and stated the case numbers and reasons. All studies had a low risk of incomplete outcome data and selectively reporting results. The 3 non‐RCT were evaluated using MINORS and the total score is 19.

### *Network of Treatment Comparisons*

**Figure 2:**
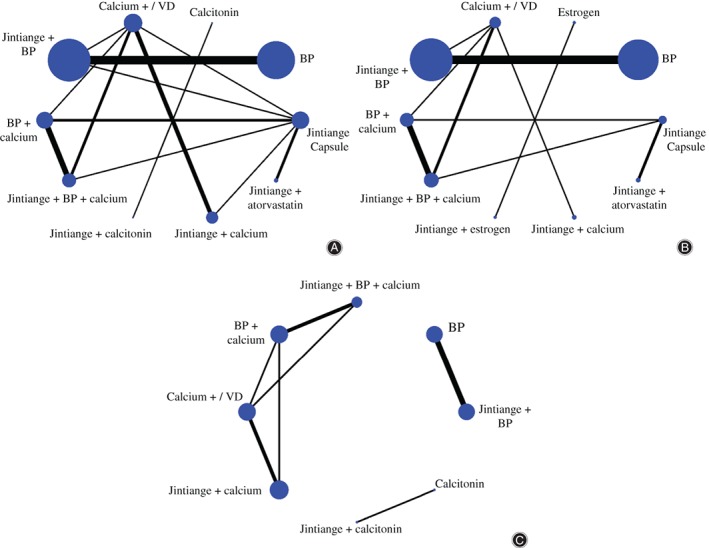
Network diagrams depicting direct evidence used in network meta‐analysis. (A) Network diagram of lumbar bone mineral density (BMD) shows 13 direct comparisons and 32 indirect comparisons. (B) Network diagram of BMD of femoral neck shows 9 direct comparisons and 36 indirect comparisons. (C) Network diagram of the visual analog pain score (VAS) shows 7 direct comparisons and 21 indirect comparisons. Lines between two nodes mean there is direct evidence between two interventions; line thickness corresponds to the number of studies and the size of the nodes represents the total sample size of the treatments.

For the BMD of the lumbar vertebra (20 studies), the results contain 10 treatments, including 13 direct comparisons and 32 indirect comparisons. For the BMD of the femoral neck (16 studies), the results contain 10 treatments, including 9 direct comparisons and 36 indirect comparisons. For the VAS (9 studies), the results contain 8 treatments with 7 direct comparisons and 21 indirect comparisons. Lines between 2 nodes mean that there is direct evidence between 2 interventions; line thickness corresponds to the number of studies: and the size of the nodes represents the total sample size of the treatments (Fig. 2).

### *Inconsistency Test Results*

For BMD of the lumbar vertebra, the interventions formed four triangle loops and two quadrangle loops. The inconsistency factors range from 0.01 to 1.12; three closed loops’ starting point is “0,” indicating that the direct and indirect evidence are consistent in these loops. In contrast, the inconsistency of evidence in another three closed loops is statistically significant.

For BMD of the femoral neck, the interventions formed two triangle loops. The inconsistency factors are 0.43 and 0.33. All loops show good consistency.

**Figure 3:**
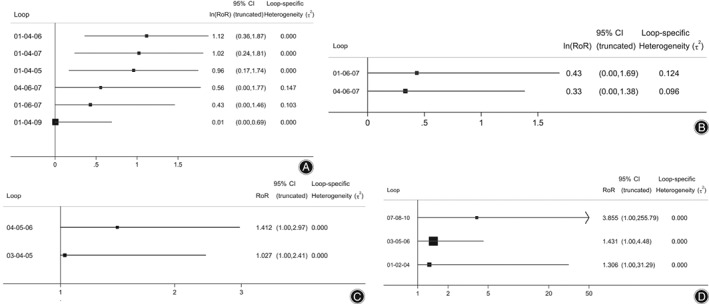
(A) For lumbar bone mineral density (BMD), the inconsistency test indicated that there are 3 closed loops showing significant inconsistency. Six loops are Jintiange capsule—calcium +/VD—BP + calcium, Jintiange capsule—calcium +/VD—Jintiange + BP + calcium, Jintiange capsule—calcium +/VD—Jintiange + BP, calcium +/VD—BP + calcium—Jintiange + BP + calcium, Jintiangecapsule—BP + calcium—Jintiange + BP + calcium, Jintiange capsule—calcium +/VD—Jintiange + calcium. (B) For the BMD of the femoral neck, the inconsistency test indicated that all closed loops showed good consistency. Two closed loops are Jintiange—BP + calcium—Jintiange + BP + calcium, calcium +/VD—BP + calcium—Jintiange + BP + calcium. (C) For the visual analog pain score (VAS), the inconsistency test indicated that all loops show significant inconsistency. Two loops are BP + calcium–calcium +/VD—Jintiange + calcium, Jintiange + BP + calcium—BP + calcium–calcium +/VD.

For the VAS, the interventions formed two triangle loops, with inconsistency factors of 1.412 and 1.027. All loops show significant inconsistency (Fig. 3).

### *Small Sample Effects and Publication Bias*

**Figure 4:**
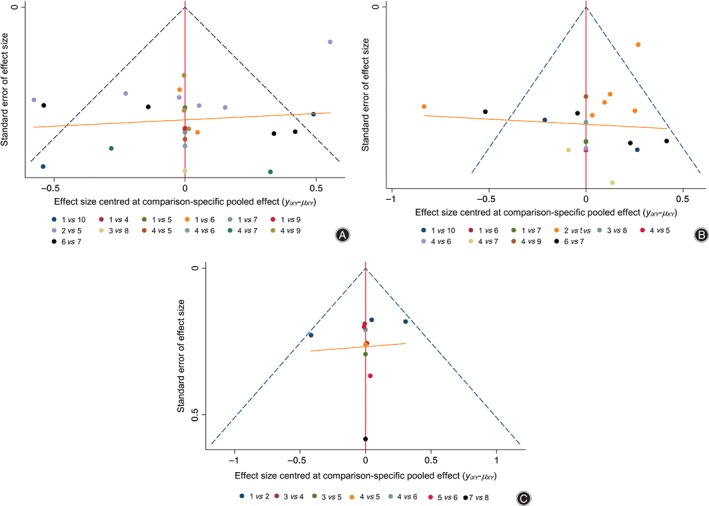
Comparison‐adjusted funnel plot of lumbar bone mineral density (BMD) (A), BMD of the femoral neck (B) and the visual analog pain score (VAS) (C). The results of BMD of lumbar vertebra and femoral neck have small sample effects, while the funnel of the VAS is symmetrical, indicating a small chance of having small sample effects. Note: 1 to 10 in Fig. 1A represent Jintiange capsule, BP, calcitonin, calcium +/VD, Jintiange + BP, BP + calcium, Jintiange + BP + calcium, Jintiange + calcitonin, Jintiange + calcium, Jintiange + atorvastatin; 1 to 10 in Fig. 1B represent Jintiange capsule, BP, estrogen, calcium +/VD, Jintiange + BP, BP + calcium, Jintiange + BP + calcium, Jintiange + estrogen, Jintiange + calcium, Jintiange + atorvastatin; 1 to 8 in fig. 1C represent Jintiange + BP, BP, Jintiange + BP + calcium, BP + calcium, calcium +/VD, Jintiange + calcium, Jintiange + calcitonin, calcitonin.

Interventions with small sample effects and publication bias were detected by comparison‐adjusted funnel plot. The points with different colors represent different direct comparisons, and the number of points with the same color represents the number of the corresponding comparison. If the funnel is symmetrical, there are no significant small sample effects and publication bias. Figure 4 shows that for the BMD of the lumbar vertebra and femoral neck, there are four and three points outside the funnel, respectively, indicating small sample effects. The funnel of the VAS is basically symmetrical, indicating that there is a small chance of small sample effects or publication bias.

### *Improvement of Bone Mineral Density of the Lumbar Vertebra*

**Figure 5:**
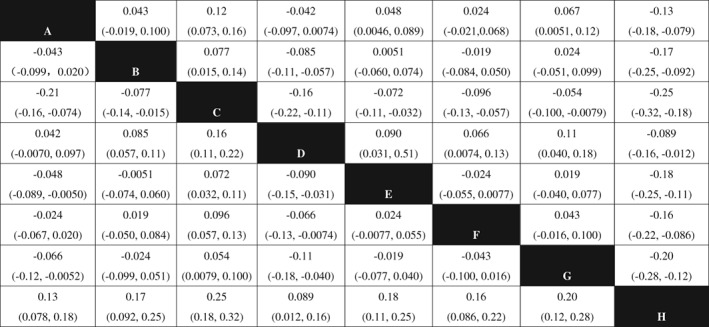
Network meta‐analysis results of lumbar BMD after using included treatments. Note: A, Jintiange capsule; B, bisphosphonate; C, calcium +/VD; D, Jintiange+BP; E, BP+calcium; F, Jintiange+BP+calcium; G, Jintiange+ calcium; H, Jintiange+atorvastatin. The data represent the MD (95%CI) value comparing the treatment in line with treatment in row. If the 95% CI of MD does not include 0 (*P* < 0.05) and MD > 0, it is considered line treatment is statistically superior than row treatment; If the 95% CI of MD include 0 (*P* > 0.05) and MD < 0, it is considered line treatment is statistically inferior than row treatment.

The NMA of lumbar BMD included 8 treatments: Jintiange capsule, BP, calcium +/VD, Jintiange + BP, BP + calcium, Jintiange + BP + calcium, Jintiange + calcium, andJintiange + atorvastatin. The results of the NMA show that the efficacy of two treatments (calcium +/VD and Jintiange + atorvastatin) have statistical significance when compared with the rest of the included treatments. The efficacy of calcium +/VD is significantly lower than that of all other treatments, and for Jintiange + atorvastatin is higher than that all other treatments. Using Jintiange capsules alone is better than five treatments, and three of them are statistically significant: Jintiange capsule *vs* calcium +/VD (MD = 0.12, 95% CI: 0.073–0.16), Jintiange capsule vsBP + calcium (MD = 0.048, 95% CI: 0.0046–0.089), Jintiange capsule *vs* Jintiange + calcium (MD = 0.067, 95% CI: 0.0051–0.12) (Fig. 5).

### *Improvement of Bone Mineral Density of the Femoral Neck*

**Figure 6:**
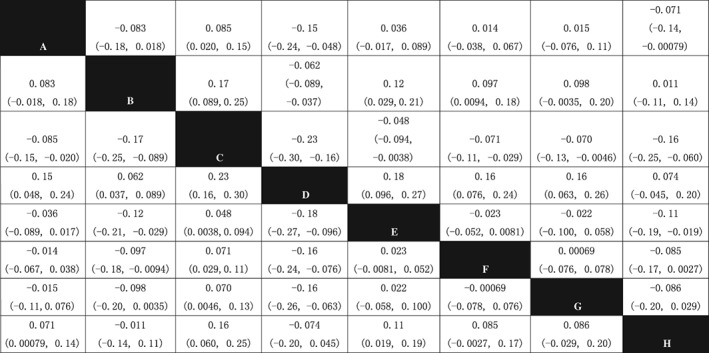
Network meta‐analysis results of BMD of femoral neck after using included treatments. Note: A, Jintiange capsule; B, bisphosphonate; C, calcium +/VD; D, Jintiange+BP; E, BP+calcium; F, Jintiange+BP+calcium; G, Jintiange+ calcium; H, Jintiange+atorvastatin. The data represent the MD (95%CI) value comparing the treatment in line with treatment in row. If the 95% CI of MD does not include 0 (*P* < 0.05) and MD > 0, it is considered line treatment is statistically superior than row treatment; If the 95% CI of MD include 0 (*P* < 0.05) and MD < 0, it is considered line treatment is statistically inferior than row treatment.

The NMA of the BMD of the femoral neck included 10 treatments: Jintiange capsule, BP, estrogen, calcium +/VD, Jintiange + BP, BP + calcium, Jintiange + BP + calcium, Jintiange + estrogen, Jintiange + calcium, and Jintiange + atorvastatin. The results of the NMA show that the efficacy of calcium +/VD is significantly lower when compared with the rest of the included treatments. In all comparisons that have statistical significance, Jintiange + BP and Jintiange + atorvastatin have better efficacy than other treatments, and no significant difference was found between these two treatments. The efficacy of a single Jintiange capsule is better than for 4 treatments, and one comparison has statistical significance, which is Jintiange capsule *vs* calcium +/VD (MD = 0.085, 95% CI: 0.020–0.15) (Fig. 6).

### *Improvement of visual analog pain Score*

**Figure 7:**
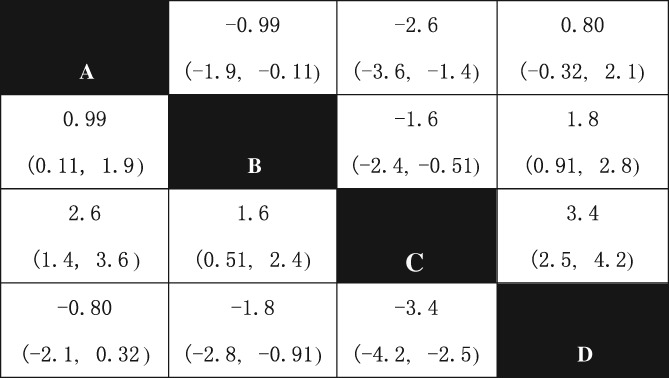
Network meta‐analysis results of VAS score after using included treatments. Note: A, Jintiange+BP+calcium; B, BP+calcium; C, calcium +/VD; D, Jintiange+ calcium. The data represent the MD (95%CI) value comparing the treatment in line with treatment in row. If the 95% CI of MD does not include 0 (*P* < 0.05) and MD > 0, it is considered line treatment is statistically superior than row treatment; If the 95% CI of MD include 0 (*P* < 0.05) and MD > 0, it is considered line treatment is statistically inferior than row treatment.

The NMA of the VAS included 4 treatments: Jintiange + BP + calcium, BP + calcium, calcium +/VD, and Jintiange + calcium. Except for Jintiange + BP + calcium *vs* Jintiange + calcium, all other comparisons have statistical significance. The score of calcium +/VD is higher than that of all other included treatments; thus, it has the worst effect in terms of pain relieve (Fig. 7).

### *Rank of Efficacy of Included Treatments*

To predict the efficacy of each treatment, SUCRA curves were drawn. For the lumbar BMD (the rank is higher when the BMD value is higher), Jintiange + atorvastatin ranks first (area under the curve = 89.8%) and calcium +/VD ranks last. For the femoral neck BMD, Jintiange + BP ranks first (area under the curve = 88.2%), and calcium +/VD ranks last. For the VAS (the rank is higher when the VAS is lower), Jintiange + calcium (area under the curve = 83.4%), indicating the best efficacy in regards to relieving pain; calcium +/VD ranks last, with the worst efficacy (Table 2).

**Table 2:** Rank of efficacy of included treatments

Treatment methods	BMD of lumbar vertebra		BMD of femoral neck		VAS	
	SUCRA value	Rank	SUCRA value	Rank	SUCRA value	Rank
Jintiange capsule	65.30%	3	48.60%	5	—	—
Jintiange + physiotherapy + calcium	—	—	—	—	—	—
Jintiange + calcitonin	53.40%	5	—	—	—	—
Jintiange + calcium	29.10%	9	40.10%	8	83.4%	1
Jintiange + Estrogen	—	—	56.00%	4	—	—
Jintiange + atorvastatin	89.80%	1	71.60%	3	—	—
Jintiange + BP + calcium	53.60%	4	41.70%	7	62.8%	2
Jintiange + BP	78.10%	2	88.20%	1	59.4%	3
physiotherapy + calcium	—	—	—	—	—	—
calcitonin	41.30%	6	—	—	—	—
calcium +/VD	11.70%	10	11.00%	10	18.8%	6
Estrogen	—	—	44.10%	6	—	—
BP + calcium	36.40%	8	26.30%	9	41.1%	4
BP	41.20%	7	72.60%	2	34.6%	5

BP, bisphosphonate; SUCRA, surface under the cumulative ranking; —, no report. For BMD (bone mineral density), the rank is upper when the value is higher; For VAS, the rank is upper when VAS is lower.

### *Safety of the Included Treatments*

#### *Incidence of Adverse Event*

Fourteen studies reported adverse events. One study (n = 32) reported using a single Jintiange capsule to treat osteoporosis, and no adverse events were found. Four studies (n = 257) reported on single use of BP, and the total number of adverse events was 28, with an incidence of 10.9%. Six studies (n = 385) reported using calcium +/VD, and the total number of adverse events was 35, with an incidence of 9.1%. Four studies (n = 249) reported using combined therapy of Jintiange capsules and BP, and there were 23 cases of adverse events, with an incidence of 9.2%. Four studies (n = 215) reported using BP plus calcium; 27 cases of adverse events were found, with an incidence of 12.6%. Four studies (n = 197) reported combined therapy of Jintiange capsules, BP and calcium; the total number of adverse events was 25, with an incidence of 12.7%. Five studies (n = 415) reported combined therapy of Jintiange capsules and calcium, and the total number of adverse events was 17, with an incidence of 4.1%.

#### *Rank of Adverse Event Incidence*

The rank of incidence of adverse events is (from high to low): Jintiange + BP + calcium, BP + calcium, BP, Jintiange + BP, calcium +/VD, Jintiange + calcium, and Jintiange capsule. When using bisphosphonate alone or in combination with other treatments, higher incidences of adverse events were found.

## Discussion

Jintiange is a biomimetic drug made of artificial tiger bone. It was listed as an effective treatment in the guidelines for the treatment of osteoporotic fractures in 2017,^2^ and some large clinical trials have demonstrated that it has good effects on osteoporosis^24^, ^42^. Primary studies and regular meta‐analysis can only be used for direct comparisons; however, to obtain comprehensive insight into the relative efficacy of the interventions, we had to rely on indirect comparisons. This study thus for the first time applied NMA to synthesize all evidence related to Jintiange capsules and Jintiange combined with other therapies, allowing a coherent comparison of the efficacy of relevant interventions in the treatment of osteoporosis. This study includes a total of 31 studies, with 4330 participants and 14 treatment methods. The main results indicated that: for improving the BMD of lumbar and femoral neck, the Jintiange combined atorvastatin and Jintiange combined bisphosphonates have the best efficacy; for relieving pain, Jintiange combined calcium has the best efficacy; Calcium combined with/without VD has the least effect for both BMD and pain relief among all regimens.

### *Bone Mineral Density of Vertebra and Femoral Neck*

In recent years, the incidence rate of osteoporotic fracture has been on the rise. The term osteoporosis was used for individuals in this population who have experienced a low‐trauma hip fracture and for those who have osteopenia by BMD who sustain a low‐trauma vertebral, proximal humerus, pelvis, or, in some cases, distal forearm fracture^43^. Among these fractures, the vertebra is the most frequently occurring site, and hip fracture is the most serious type, significantly affecting patients’ quality of life and bringing huge economic burden to them^2^, ^44^. Consequently, the BMD of the vertebra and femoral neck were used as the primary outcome measure in this study. Bone pain, mostly lower back pain, is the most common syndrome for osteoporosis. This study thus took the VAS as one of the secondary outcome measures.

The results of this study showed that Jintiange capsules combined with atorvastatin and with BP had the best efficacy in terms of improvement of the BMD of the lumbar and femoral neck, respectively, and using Jintiange alone was also superior to some other regimens. These results are consistent with many previous studies. Zhang^7^ applied an enzyme digestion method to culture osteoblasts, and the author observed that the alkaline phosphatase (ALP) and osteocalcin increased by adding more Jintiange and urine pyridine decreased, which illustrates that Jintiang can accelerate osteoblasts. Fan and Li^45^established an osteoporotic animal model using retinoic acid and investigated the effect of biomimetic tiger bone on osteoporosis. They found that this drug can effectively improve the trabecular bone structure, increase the activity of ALP and decrease the activity of tartrate‐resistant acid phosphatase, which means it could accelerate osteoblasts and inhibit osteoclasts.

### *Efficacy in Relieving Pain*

The best method of relieving pain in this study was Jintiange capsules combined with calcium; this may result from the analgesic effect of tiger bone. Previous studies support this result. Hai^9^conducted acetic acid writhing and electric shock on mice tails to test the analgesic effect of tiger bone, and the results showed that it can effectively relieve pain. Se *et al.* 46also demonstrated that tiger bone can enhance the pain threshold and prolong the latent period of pain response by conducting the hot plate test and the acetic acid writhing test on mice.

### *Incidence of Adverse Events*

Jintiange capsules are an oral medication that has the advantage of good compliance and safety, with no obvious effect on hepatic and renal function^6^. Because many studies did not report adverse events or report incomplete data and was is not possible to contact the authors, we did not conduct network meta‐analysis on adverse events, and data were qualitative combined. The present study took adverse events as one of secondary outcome measures, and the results are similar to those of previous studies. According to our analysis, using Jintiange capsules alone and Jintiange combined with calcium have the lowest incidence rate of adverse events. Regimens that include bisphosphonates are inclined to be associated with more adverse events.

### *Limitation of the Study*

The study has some limitations: (i) adverse events of all regimens were not included in the NMA; (ii) there is concern about the methodological quality in the include studies given that all RCT did not describe allocation concealment or blinding, which may influence the final conclusions; (iii) inconsistency for three loops in lumbar BMD and two loops in the VAS had statistical significance; (iv) publication bias was observed in studies that report the BMD of the lumbar and femoral neck.

### *Conclusion*

This NMA provides evidence that Jintiange capsules and Jintiange combined with some therapies can effectively increase the BMD, relieve pain, and lower the incidence of adverse events, and could be a good choice for treating osteoporosis with high effectiveness and safety. Due to the limitations of this study, higher‐quality RCT are needed to confirm these results.
